# Presenting Your Best Self: How Physician Appearance Influences Patient Perceptions and Impacts Women in Medicine

**DOI:** 10.1089/whr.2024.0087

**Published:** 2025-01-08

**Authors:** Andrea Lopez-Ruiz, Lopa Misra, Lindsey Lamb, Julia Files, Sonal Haerter, Lisa Marks, Neera Agrwal

**Affiliations:** ^1^ Mayo Clinic Alix School of Medicine, Scottsdale, Arizona, USA.; ^2^ Department of Anesthesiology, Mayo Clinic, Phoenix, Arizona, USA.; ^3^ Arizona College of Osteopathic Medicine, Glendale, Arizona, USA.; ^4^ Department of Internal Medicine, Mayo Clinic, Phoenix, Arizona, USA.; ^5^ Creighton University School of Medicine, Phoenix, Arizona, USA.; ^6^ Library Services, Mayo Clinic, Phoenix, Arizona, USA.

**Keywords:** women physicians, gender bias, patient perception, patient adherence

## Abstract

**Introduction:**

Trust and rapport between patients and physicians form the cornerstone of effective medical practice. A key factor influencing this trust is the patient’s perception of the physician’s appearance. Women physicians often face more rigorous expectations concerning their physical appearance compared to men physicians, creating a need to balance traditional professional attire with maintaining femininity and individuality. This review explores the unique challenges women physicians encounter in presenting a professional image.

**Methods:**

A narrative review was conducted using PubMed and Google Scholar to identify studies addressing both patient and physician perceptions of physician appearance. The search was limited to studies conducted in the United States and published between 2004 and 2024. Seven articles met the inclusion criteria and were selected for analysis.

**Results:**

The findings suggest that professional attire, particularly when combined with a white coat, is associated with higher perceptions of competence and professionalism. However, women physicians face ambiguity in meeting these expectations compared with men. In addition, women are more frequently mistaken for nurses, phlebotomists, or support staff based on their attire and appearance.

**Discussion:**

Overall, patients tend to prefer professional attire to physicians, but these preferences are influenced by cultural, demographic, and age-related factors. Women physicians, in particular, experience heightened scrutiny regarding their appearance and are subject to stricter norms than men physicians. This challenge is further amplified for young women trainees, who may struggle to convey professionalism due to their youthful appearance.

## Introduction

The transformative power of dress has been long recognized and embedded in cultural narratives, from fairy tales such as *Cinderella* to classical plays such as *Pygmalion* by George Bernard Shaw, later adapted into the musical *My Fair Lady.* These narratives underscore the significance of attire and overall appearance in shaping both how others perceive us and how we perceive ourselves. The influence of dress extends beyond aesthetics, encompassing a complex interplay of social and psychological factors that contribute to personal and professional identities.

The formal definition of dress, as outlined by Roach-Hinngins and Eicher, is “an assemblage of modifications of the body and/or supplements to the body.”^1^ Dress, along with personal aesthetics, serves as a tool for conveying social status, cognitive state, and group affiliation. Furthermore, the psychological impact of dress is deeply intertwined with social and cultural contexts.^2^ It not only shapes self-expression but also functions as a nonverbal communication tool, signaling one’s intentions, and professional identity.

In professions where appearance plays a crucial role in establishing credibility, women, particularly those in men-dominated fields, often face unique challenges. For women physicians, their professional attire must strike a delicate balance between competence, approachability, and femininity. Studies have shown that over half of patients place significant importance on physician attire, which in turn influences their satisfaction with medical consultations.^3^ Thus, women physicians must navigate an intricate web of expectations that their men counterparts are less frequently subjected to.

Research demonstrates that women physicians face more stringent expectations regarding their appearance compared to men physicians. A study published in JAMA Network Open revealed that patients were less likely to identify a woman wearing scrubs as a physician, highlighting a pervasive gender bias that undermines the perceived professionalism of women in medicine.^4^ Such biases manifest in various ways, including the tendency to misidentify women physicians as nurses or support staff based on their attire and overall appearance.

Beyond clothing, women are also scrutinized for their nonverbal behaviors and vocal cues. A Swiss study evaluating nonverbal communication found that women physicians were perceived as less likable when exhibiting behaviors typically associated with assertiveness, a judgment that did not apply to their men colleagues.^5^ This discrepancy suggests that women must modulate not only their attire but also their demeanor to conform to societal expectations of likability and professionalism.

The heightened scrutiny of women’s appearance in medicine was brought into sharp focus in a 2020 incident widely known as #BikiniGate. In this case, the Journal of Vascular Surgery published an article criticizing women vascular surgeons for posting vacation photos in bikinis on social media, arguing that such images were unprofessional. Although the article was retracted following widespread backlash, the incident underscored the persistent double standards applied to women in professional settings.

These social expectations create a professional double bind for women in medicine. They must conform to traditional standards of professionalism, often defined by men-centric norms, while maintaining an image that is neither too assertive nor too feminine. The cumulative effect of these conflicting expectations can lead to uncertainty regarding how to project a professional identity that aligns with both patient expectations and personal authenticity.

This narrative review aims to analyze the impact of attire on patient perceptions of women physicians. The review includes studies that examine both men’s and women’s physicians’ attire to contextualize the distinct challenges faced by women. By synthesizing findings from the existing literature, this article seeks to illuminate the complexities of professional appearance for women in medicine and provide a comprehensive overview of how attire influences perceptions of competence and professionalism.

## Methods

A literature search was conducted with the assistance of a medical librarian. Ovid Medline® was searched from 1946 to the present, along with epub ahead of print, in-process, and other nonindexed citations. Google Scholar was also searched in order to look for articles published in nonmedical journals and magazines. Search terms included business attire, business dress, professional dress, clothing, professional attire, woman, female perception, perspective, patient perspective, patient perception, physician–patient relations, or patient satisfaction.

Inclusion criteria were peer-reviewed research studies conducted in the United States that examined the relationship between physician attire and patient perception of their woman physician. Systematic reviews, meta-analyses, and narrative reviews were included if they met the inclusion criteria. We excluded any study that did not have data pertaining to the impact of dress codes on women physicians. Three authors reviewed the inclusion criteria. A total of 39 articles were initially identified as potentially relevant. We focused on articles published after 2004, due to the changes in the dress code over the decades. Seven articles met the inclusion criteria.

## Results

### Procedural specialties

Two studies specifically looked at procedural specialties (Table 1).

**Table 1.** Summary of Narrative Review Key Findings in Procedural Studies

Authors	Publication year	Setting	Study design	Sample size	Key findings
Cha et al.	2004^6^	Procedural	Cross-sectional survey study	*N* = 188	Patients do not self-report a difference in their confidence or comfort levels with their physicians based on attire. Patients report the highest comfort level with providers in scrubs with a white lab coat. Patients report the lowest comfort levels with providers in casual dress.
Jennings et al.	2016^7^	Procedural	Cross-sectional survey study	*N* = 85	Physicians in casual attire were rated poorly in all categories. Patients consider surgeons in scrubs to be the most confident.

A 2004 study at the Aultman Hospital Obstetrics and Gynecology Clinic in Ohio asked patients (*n* = 188) to rate their level of confidence and comfort with certain providers based on photographs provided.^6^ The photographs pictured men and women physicians in scrubs, casual attire (pullover sport shirt and slacks for men and blouse and slacks for women), and business attire (shirt, slacks, and tie for men and blouse and skirt for women), all with and without a white lab coat. They were also asked to self-report their preferences on physician dress. The study found that most patients did not self-report a difference in their confidence in or comfort with either sex provider based on their attire, although patients reported the highest comfort level and perception of competence when examining an image of a physician in scrubs with a white coat, and the lowest when presented with an image of a physician in casual dress. The mean confidence level in women physicians was lower in all attire categories except scrubs without a white lab coat and casual. Similarly, the mean comfort level with women physicians was lower across all attire categories except casual attire.

A 2016 study administered a three-part questionnaire (*n* = 85) to patients in an orthopedic surgical setting where patients were asked to rate images of either a man or woman surgeon wearing scrubs, business attire (business suit for men and blouse and skirt for women), casual attire (shirt and jeans for men and women), and business attire with a white lab coat on a 5-point Likert scale (*i.e.,* confidence, trustworthiness, safety, caringness, intelligence, how well the surgery would go, and how safe they would feel discussing vulnerable information).^7^ Casual attire was rated poorly in all categories for both men and women surgeons. Surgeons in business attire and white lab coats were deemed more confident than those in business attire (*p* = 0.006) and casual attire (*p* < 0.001) but not more confident than those in scrubs (*p* = 1.000). Patient ratings of women surgeons in scrubs and a white lab coat did not differ significantly. However, women surgeons pictured in a white lab coat were rated more positively in four out of seven categories compared to those in casual attire (confidence: *p* = 0.028; discussion of vulnerable information: *p* = 0.010; trustworthiness: *p* = 0.044; safety: *p* = 0.010). Scrubs also elicited higher confidence ratings than business attire (*p* = 0.042).

### Nonprocedural specialties

One study met these criteria (Table 2).

**Table 2.** Summary of Narrative Review Key Findings in Nonprocedural Studies

Authors	Publication year	Setting	Study design	Sample size	Key findings
Rehman et al.	2005^8^	Nonprocedural	Cross-sectional survey study	*N* = 400	Patients indicate that they are more willing to engage in vulnerable conversations such as discussing their social, sexual, and psychological concerns if their physician is in professional attire. Patient adherence and follow-up rates increase when their physician is wearing professional attire with a white lab coat.

In 2005, Rehman et al. distributed surveys to patients (*n* = 400) in an internal medicine outpatient clinic.^8^ The pictured physicians were a white woman, a white man, a black woman, and a black man. The four dress styles presented were (a) business attire (business suit with necktie for men and either tailored trouser or skirt for women), (b) scrubs, (c) casual dress (t-shirt and jeans for men and t-shirt and short skirt or jeans for women), or (d) professional attire (shirt, necktie, and white lab coat for men and tailored trouser or skirt with white lab coat for women). Patients reported that professional attire was more conducive to critical conversations. Respondents also reported a greater willingness to share social, sexual, and psychological concerns with a physician in professional attire. The study also found that patient adherence and follow-up rates increased when the physician was dressed professionally with a white coat. Patients shown images of women physicians indicated that they placed a greater emphasis on physician appearance compared to patients who viewed images of men physicians (*p* < 0.001). In particular, women patients placed more importance on women’s physician attire, yet men patients rated the importance of men’s and women’s physician attire fairly equally (*p* = 0.13).

### Both procedural and nonprocedural specialties

Three studies were included in this category (Table 3).

**Table 3.** Summary of Narrative Review Key Findings in Both Procedural and Nonprocedural Studies

Authors	Publication year	Setting	Study design	Sample size	Key findings
Varnado-Sullivan et al.	2019^9^	Varied	Cross-sectional survey study	*N* = 505	• Women are more likely to be identified as nurses or receptionists regardless of attire. • Showing bare legs when wearing dresses or skirts is deemed inappropriate by patients. • White lab coats did not increase ratings of professionalism and decreased ratings of humanism in providers.
Petrilli et al.	2018^3^	Varied	Cross-sectional survey study	*N* = 4062	Patients believe that their physicians’ attire during their visit is important. Formal attire with a white lab coat was rated highest by patients.
Fox et al.	2016^10^	Varied	Cross-sectional survey study	*N* = 255	A majority of patients prefer their dermatologists to wear professional attire.

A study conducted at the University of Miami in 2016 examined dermatology patients’ (*n* = 255) preference for attire in the treating physician. The majority of the survey participants self-identified as Hispanic.^10^ Results demonstrated that 73% of patients preferred professional attire, 19% preferred surgical attire, 6% preferred business attire, and 2% preferred casual attire, where casual attire was defined as jeans, business attire consisted of a suit and tie, professional attire was defined as a white lab coat and tie, and surgical attire was scrubbed. Patients, when shown images of black physicians, were more likely to specifically prefer professional attire compared to when they were shown images of white men physicians. This finding was applicable to images of both black men and women physicians.

In a study conducted between 2015 and 2016, questionnaires (*n* = 4062) were administered to patients across ten academic hospitals in the United States.^3^ Patients were shown photographs of women and men physicians in the following seven outfits: scrubs with and without a white lab coat, casual dress (collared, short-sleeved shirt, and jeans) with and without a white lab coat, formal attire (buttoned-down dress shirt and suit pants) with and without lab coat, and in a business suit. Participants were asked to rate the following characteristics of each image: knowledge, trust, care, approachability, and comfort. Most patients (53%) indicated that the attire their physician wore mattered to them. Preferences based on care settings differed. Patients rated formal attire the highest in primary care (44%) and hospital medicine (39%) settings, but rated scrubs the highest in emergency room (40%) and surgery (42%) settings. Physician attire including a white lab coat was preferred by patients, except if the physician was a surgeon. When considering surgeon attire, a white lab coat was preferred for men physicians (*p* < 0.001), but no preference for a white lab coat in women physicians was noted (*p* = 0.85).

In 2019, an online survey (*n* = 505) conducted by Varnado-Sullivan et al. in Louisiana sought to examine patient and fellow peer perception of men and women physicians from three ethnic categories (Caucasian, African American, and Indian American).^9^ A 7-point Likert scale was utilized to rate professionalism and humanism in these physician models. The models were dressed in business casual attire (pullover shirt and pants) with a white lab coat, business casual attire without a lab coat, professional attire (button-down shirt and pants) with a white lab coat, or professional attire without a white lab coat. The study concluded that business casual and professional attire were both highly rated, a name tag was felt to be very important, and physicians were rated as warmer and kinder when not wearing a white coat. When rating women’s physician attire, all respondents stated that when wearing dresses and skirts, bare legs were inappropriate. Caucasian women physicians received the highest ratings when in professional attire with a white coat. Women physicians were more likely to be labeled as receptionists or nurses regardless of clothing. Conversely, men were more likely to be identified as physicians or lab assistants. Overall, Caucasian women physicians were rated highest in humanism.^9^ Physicians older than 35 years of age received higher humanism ratings when wearing business casual attire. However, despite a lower humanism rating with professional attire, respondents stated they still preferred professional attire over business casual attire.

### Unspecified medical care settings

One study was reported in this category (Table 4).

**Table 4.** Summary of Narrative Review Key Findings in Both Unspecified Care Setting Studies

Authors	Publication year	Setting	Study design	Sample size	Key findings
Xun et al.	2021^4^	Unclear	Cross-sectional survey study	*N* = 487	Regardless of attire, women models received lower professionalism ratings than men models. Women models were more likely to be identified as nonphysician healthcare staff compared to men models who were more likely to be identified as physicians.

Xun et al. published a study in 2020 comparing traditional professional attire to casual attire, including the impact of outerwear, consisting of either a white lab coat, fleece jacket, or softshell jacket, on either scrubs.^4^ Respondents (*n* = 487) were instructed to rate men and women models dressed in business attire or scrubs with an outer layer of a white coat, fleece jacket, or softshell jacket on a 6-point Likert scale. A rating of one indicated the least experience, professionalism, and friendliness, and a rating of six indicated the most experience, professionalism, and friendliness. The models in white coats were considered the most experienced (4.9), while those in fleece and softshell jackets were considered less experienced (3.1). In terms of regional variations in preference for physician attire, it was found that a fleece jacket worn with scrubs was associated with reduced professionalism in all areas of the United States except the Western United States.^4^ Regardless of outerwear, women models were rated less professional than their men counterparts, even when wearing business professional attire (*p* < 0.001). Men models were more likely than women models to be perceived as physicians, whereas women models were more likely to be labeled nurses, phlebotomists, and technicians (*p* < 0.001).

## Discussion

The purpose of this review was to examine the current literature on the challenges faced by women physicians in projecting a professional image to patients. While the intention was to analyze studies specifically focusing on women physicians, the scarcity of research directly addressing this issue required us to extrapolate gender-specific data from broader studies on physician attire. This gap in the literature underscores the need for more focused research on patient perceptions of women physicians and their professional presentation.

The findings indicate that, in general, professional attire is preferred by patients, with the white coat being the most favored option across most settings.^1,2,6–8^ However, one study noted that the white coats decreased the perceived humanism of physicians.^9^ Casual attire, on the contrary, was consistently rated poorly for both surgical and nonprocedural contexts.^5,10^ Women physicians experience unique challenges related to patient expectations and biases, in that they are more likely to be misidentified as nurses or ancillary staff due to stereotypes and preconceptions, highlighting a distinct professional obstacle rooted in gender-based assumptions.^5,9,10^

A critical factor influencing these biases is the rigid appearance norms imposed on women compared with men. The ideal image of women portrayed in society tends to adhere to a narrow and often unattainable standard, whereas the expectations for men are more flexible and forgiving.^11^ This disparity has tangible consequences, as women feel pressured to conform to idealized norms of appearance to be perceived as competent. The psychological impact of these expectations is well-documented; for instance, Frederickson et al. found that women wearing a swimsuit performed worse on cognitive tasks compared to those wearing a sweater, illustrating how appearance can influence self-perception and performance.^12^ Conversely, research by Adam and Galinsky demonstrated that wearing a white coat led to enhanced cognitive performance in tasks requiring sustained attention.^13^ These findings suggest that attire not only affects how others perceive women but also influences how women perceive themselves, potentially impacting their confidence and professional identity.

Provocative clothing is a particular area of concern, as it can lead to negative perceptions of competence and professionalism. Provocative attire, characterized by revealing or suggestive styles, has been associated with stereotypes of sexual availability and lower professional credibility. Research outside the medical field, such as that by Howlett et al., found that women wearing such clothing were judged more negatively in terms of competence and permissiveness, regardless of the evaluator’s gender.^14^ Although no studies have explicitly examined this issue among women physicians, the #bikinigate incident involving women vascular surgeons in 2020 illustrates that such biases are present in the medical profession as well.

Another factor influencing professional perception is the use of identification badges. A study conducted at the Mayo Clinic revealed that women physicians experienced a significant reduction in misidentification as support staff when wearing name tags clearly labeled as “Doctor”.^15^ Prior to this intervention, 81.8% of women residents were misidentified as support staff weekly, but this figure dropped to 18.2% within two months of using the labeled badges. This finding indicates that visual cues beyond attire, such as name tags, can play a critical role in establishing professional identity and reducing gender-based misconceptions.

The white coat, often considered the most emblematic symbol of the medical profession, has a complex history. Initially popularized in the late 19th century as a symbol of cleanliness and professionalism, it remains deeply ingrained in the medical identity today.^16^ Despite its symbolic significance, recent studies have raised concerns about its role in infection control due to potential contamination. While the data directly linking medical white coats to healthcare-associated infections are inconclusive at best, this issue remains a point of consideration in discussions about the balance between appearance and safety in medical settings.^17^

Ultimately, the data suggest that women physicians face greater ambiguity and scrutiny regarding their professional presentation compared to men colleagues. They must navigate a delicate balance between appearing competent and approachable, often within a narrow window of acceptable dress and behavior. The cornerstone of medical practice rests on the establishment of trust and rapport during patient–physician interactions. Central to this trust is the perception patients hold of their physician’s professional appearance. Physician attire plays a significant role in shaping these perceptions. Within the high-stress world of medicine, women also want to maintain their individuality. This dilemma is further compounded for young women physicians, who may struggle with patient perceptions of inexperience or lack of authority due to their age and gender. The cumulative effect of these challenges may contribute to higher levels of burnout and emotional stress among women physicians, potentially impacting their long-term career satisfaction and retention in the field.

The perception of women physicians’ dress is one of many barriers they face in the healthcare profession. Addressing this issue is critical not only for individual well-being but also for promoting a more equitable professional environment. Leadership in healthcare institutions should prioritize the development of guidelines that acknowledge and mitigate the unique challenges faced by women physicians. Programs that support professional development for women should emphasize both competence and individuality, allowing women to express their personal style without compromising their professional identity.

Future research should focus on patient perceptions of women physicians’ attire to better understand how specific elements of dress influence professional evaluations. Longitudinal studies examining the impact of attire on career progression, burnout, and patient trust would be particularly valuable. In addition, research should explore the role of institutional culture in shaping expectations for women’s appearance and how these expectations can be modified to foster a more inclusive professional environment.

The conclusions drawn from this review are limited by the availability and scope of the studies included. Potential biases related to the attractiveness of models used in surveys, the lack of consistent definitions of attire categories, and variations in sample sizes may have influenced the findings. All the summarized studies were conducted in the United States, which limits the generalizability of the conclusions to other countries, as white lab coats may not be the preferred attire in all cultures. Furthermore, many studies did not include scrubs and white coats as distinct categories, limiting the ability to fully assess their relative impact on patient perceptions.

Overall, while this review highlights significant gaps in the literature, it provides a foundation for future research on the complexities of professional appearance for women physicians and its impact on patient interactions and career development. Addressing these gaps will be essential in creating a more supportive and equitable environment for women in medicine.

## Conclusion

The existing literature highlights a multifaceted relationship between attire, gender, and professional identity, creating distinct challenges for women in medicine. This complex interplay may contribute to increased burnout among women physicians, partly due to the heightened scrutiny and conflicting expectations related to their appearance. Future research should prioritize establishing evidence-based guidelines that address these gender-specific challenges and provide clear recommendations for professional attire. Additionally, leadership initiatives should support programs that promote professional development for women while respecting and encouraging their individuality.
